# Muscle strength during pregnancy and postpartum in adolescents and adults

**DOI:** 10.1371/journal.pone.0300062

**Published:** 2024-03-27

**Authors:** Maria Luiza da Silva Santos, Sabrina Gabrielle Gomes Fernandes Macêdo, Juliana Fernandes, Catherine M. Pirkle, Saionara M. A. Câmara

**Affiliations:** 1 Faculty of Health Sciences of Trairi, Federal University of Rio Grande do Norte (UFRN), Santa Cruz, Brazil; 2 Postgraduate Program in Physiotherapy, Federal University of Rio Grande do Norte, Natal, Rio Grande do Norte, Brazil; 3 Postgraduate Program in Physiotherapy, Federal University of Pernambuco, Recife, Pernambuco, Brazil; 4 Office of Public Health Studies of University of Hawaiʻi at Mānoa, Honolulu, Hawaii, United States of America; 5 Postgraduate Program in Rehabilitation Sciences, Federal University of Rio Grande do Norte, Santa Cruz, Rio Grande do Norte, Brazil; The University of British Columbia, CANADA

## Abstract

Adolescent childbirth is associated with worse physical function over the long-term. Differential loss of muscle strength during pregnancy and postpartum for adolescents compared to adults may be one explanation for this, but research examining these differences is lacking. The objective of this study as to assess hand grip strength and hip adduction muscle strength in adolescents and adults during pregnancy and postpartum. A prospective cohort study was carried out with adolescent (13 to 18 years) and adult (23 to 28 years) primigravid women. Assessments were performed at three timepoints: before the 16^th^ gestational week, during the third trimester, and between the fourth and sixth week postpartum. Hand grip strength (continuous and muscle weakness if ≤ 20.67 kgf) and hip adductor measures (continuous and muscle weakness if ≤ 13.8 kgf) were assessed using dynamometry. Generalized estimating equations modelled longitudinal relationships between muscle weakness and age group. More adolescents had hip adductor weakness than adults in the third trimester of pregnancy (62.5% vs. 31.8%, p < 0.005), which was corroborated by the longitudinal analyses. For all women, there were higher odds of hip adductor weakness in the third trimester (OR = 4.35; p< 0.001) and postpartum (OR = 9.45; p < 0.001) compared to the 16^th^ gestational week. No significant difference in HGS was observed between age groups or across the different timepoints. The higher proportion of hip adductor weakness among adolescents may indicate a need for resistance training during and after pregnancy and physical therapy if weakness or injury is noted.

## Introduction

Adolescence is a life-stage characterized by rapid growth and substantial changes across body systems [1]. It is a particularly sensitive period during which events can contribute to long-term poor health [1]. Increasing evidence indicates that pregnancy during adolescence is harmful to later-life health, as measured by a greater likelihood of chronic disease, poor physical function and frailty [2–4].

The mechanisms between pregnancy during adolescence and impaired physical function at older ages are not fully understood. Reduced muscle mass and strength reserves before pregnancy, as well as differential loss of muscle strength during pregnancy and postpartum for adolescents compared to adults are possible explanations.

During pregnancy, the progressive increase in ligament laxity facilitates the adaptation of the pelvic structure to the uterus and promotes the remodeling of connective tissues, contributing to pelvic instability [5]. As pregnancy progresses, body weight increases, the uterus expands, and the abdomen dilates, bringing the gravity center forward and increasing pressure on the pelvic floor [6]. Evidence from animal models suggest that there are adaptations in muscle-tendon morphology in response to the hormonal and mechanical changes from pregnancy [7, 8]. Pelvic floor muscles adapt increasing sarcomere length and with sarcomerogenesis [7], type I fibers of lower limb muscles become smaller in diameter and quantity during pregnancy and postpartum, with a reduction in tendon stiffness during pregnancy that may persist through lactation [8]. These are likely to affect muscle performance and impair mobility. It has been suggested that they may be harmful when physiological development is incomplete (e.g., during adolescence) [3, 9]. Because adolescents have not reached full musculoskeletal development, their muscles, tendons and ligaments may not be prepared to support pregnancy and childbirth without damage, and this could lead to greater and lasting impact on the musculoskeletal system. Additionally, when pregnancy occurs with less muscular reserve, as in adolescence, the physiological changes of pregnancy may contribute to muscle weakness more readily, which may impact body functioning and development. However, research examining differences in these musculoskeletal aspects between pregnant adolescents versus adults is lacking.

Most studies that have analyzed the relationship between adolescent pregnancy and physical performance were retrospective and conducted with middle-aged and older women [3, 10]. They found that those who gave birth at 18 years or younger had worse physical performance and suggest that adolescent pregnancy may permanently affect the musculoskeletal system and impair physical functioning as the woman ages [3, 10].

However, musculoskeletal changes during pregnancy and differences between adolescent and adult pregnant women are not well established. In this sense, understanding if muscle strength during pregnancy and the postpartum period varies in adolescents and adults may elucidate relationships between early pregnancy and worse physical performance at older ages and support prevention strategies. Therefore, this study aimed to assess and compare muscle weakness in adolescents and adults during pregnancy and postpartum.

## Methods

### Study type and location

This is a prospective cohort study called Adolescence and Motherhood Research (AMOR) [11]. The AMOR project aimed to establish a research platform to investigate adolescent pregnancy and adverse health consequences in later life. This study involved pregnant women living in a low-income region in northeastern Brazil. Reporting follows the STROBE guidelines [12].

### Population and sample

The sample consisted of 50 pregnant adolescents (13 to 18 years) and 50 pregnant adults (23 to 28 years) recruited from primary health units, community centers, and through contact with the nurses of family health teams as well as community health agents. Pregnant women could also directly contact researchers to volunteer for the study.

A sample size of 100 was established because it is considered sufficient to assess psychometric properties of epidemiological instruments [13], which was one of the main objectives of the AMOR project.

**Fig 1:**
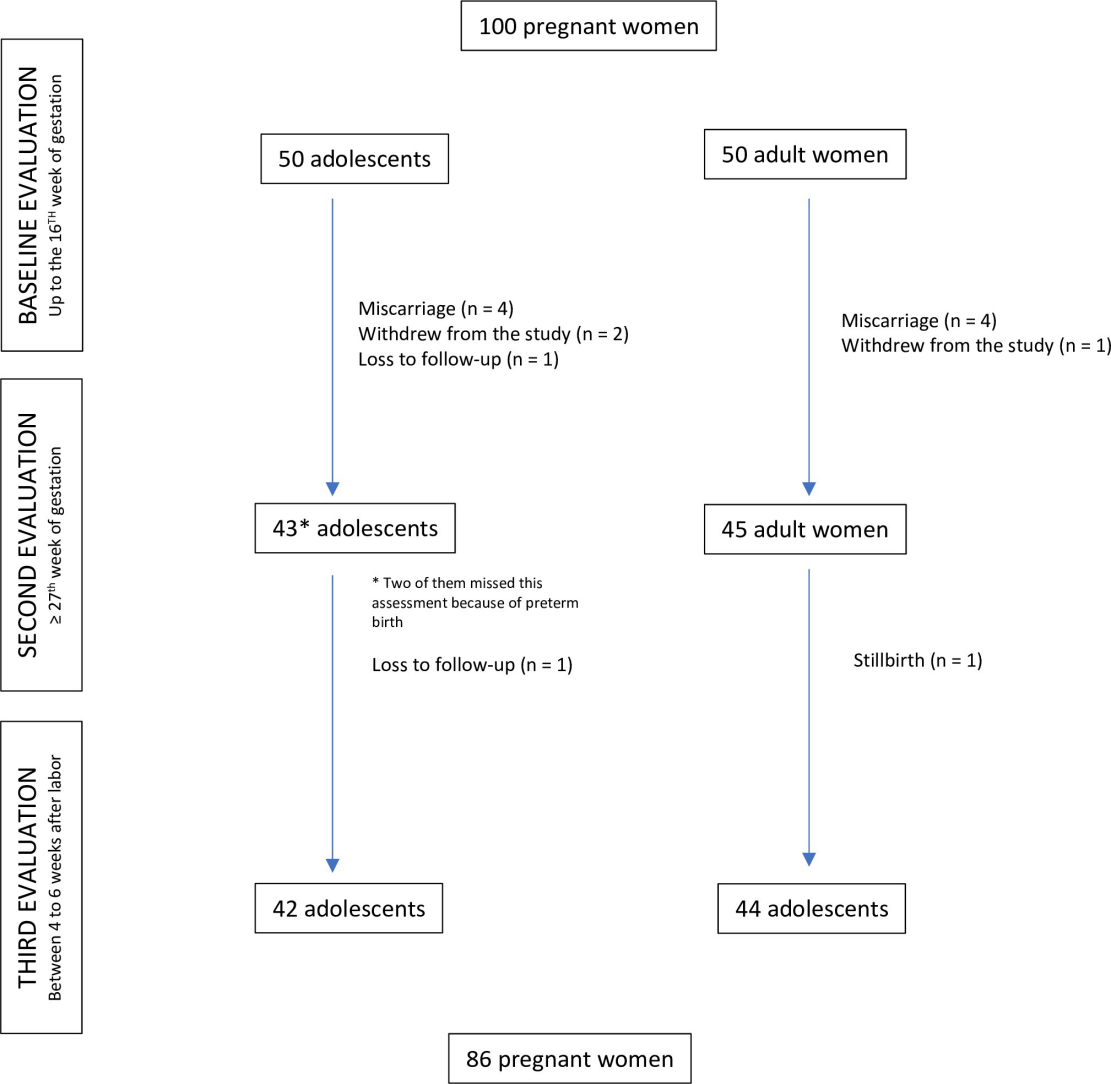
Flowchart of the sample according to age group from the Adolescence and Motherhood Research (AMOR) project in the trairi region, Rio Grande do Norte, Brazil, 2017–2019.

Overall, 100 participants completed the first assessment and 86 completed all three assessments. Fig 1 presents a flow-chart of participation over the duration of the study.

### Eligibility criteria

Pregnant adolescents (13 to 18 years) and adults (23 to 28 years), primigravid, and not exceeding the 16^th^ gestational week in the first assessment, were included. Maximum age of adolescents was 18 at baseline, since the risks related to childbirth significantly reduce after this age [14]. The adult group was also age-delimited based on the range associated with lower risk of chronic diseases related to pregnancy [15]. Exclusion criteria were diagnosis of psychiatric or chronic diseases (e.g., diabetes mellitus, heart diseases, human immunodeficiency virus or acquired immune deficiency syndrome, cancer, tuberculosis, epilepsy, and lupus) before pregnancy and prolonged use of antidepressant and anxiolytic drugs (n = 1).

### Ethical aspects

The study was approved by the human research ethics committee of the Federal University of Rio Grande do Norte and followed the resolution 466/2012 of the National Health Council. All pregnant adults and legal guardians of pregnant adolescents signed an informed consent form, following Brazilian ethical recommendations. Adolescent participants also signed an assent form (approval: 2.151.554).

### Data collection

Rigorously trained interviewers applied a structured questionnaire between July 2017 and January 2019 [11]. Participants were assessed at three timepoints: before the 16^th^ gestational week, during the third trimester of pregnancy (27^th^ to 41^st^ gestational week), and between the fourth and sixth week postpartum.

#### Sociodemographic variables

At baseline age, race/color (black, brown, or white as defined by the Brazilian census), marital status (married or stable union, dating, or without partner), and income sufficiency for basic needs (very sufficient, sufficient, or insufficient) were collected. Educational level was defined as less than elementary school or elementary school or more. In Brazil, elementary school is completed after 9 years of schooling, which most children complete by age 14.

#### Postpartum variables

These included self-reported information on type of delivery (vaginal or cesarean), episiotomy (yes or no), and number of days in the hospital after delivery.

#### Physical activity practice

Physical activity was assessed at baseline and third trimester using the following question: “Have you performed any physical exercise in the last week, for example, walking?”(yes/no).

#### Self-reported health

Self-reported health was assessed using a visual scale, akin to a thermometer, ranging from 0 to 100, in which 0 represented the worst imaginable state of health and 100 the best state. Participants drew a line on the scale according to the following question: "We would like you to indicate on this scale how good or bad your health status is today". Results were categorized considering the 20^th^ percentile of participant values as the cutoff point for "good" (≥ 70) or "moderate/bad" (< 70). Data from the first assessment were considered for sample characterization.

#### Anthropometric measurements

Weight and height were assessed at baseline to calculate body mass index (BMI) and classified according to the World Health Organization (underweight, < 18.5 kg/m^2^; normal weight, ≥ 18.5 and < 25 kg/m^2^; overweight, ≥ 25 and < 30 kg/m^2^; or obese, ≥ 30 kg/m^2^) [16].

#### Muscle strength

Handgrip and hip adductor strength were measured at all three assessments. Handgrip strength was assessed using a Saehan® dynamometer, following the North American Society of Hand Therapists guidelines [17]. Volunteers were seated with shoulder adducted and in neutral rotation, elbow flexed to 90°, and forearm and wrist in neutral position or with slight wrist extension. Three maximal isometric contractions were performed three times, for five seconds, with one minute of rest between them. The mean of the three measurements was used for data analysis [18]. Intraclass correlation coefficients (two-way mixed effects, absolute agreement, single measurement) showed that the three attempts were highly correlated in each time-point (Baseline: ICC = 0.88, 95%CI = 0.84–0,91, p<0.001; 1^st^ follow-up: ICC = 0.91; 95%CI = 0.88–0,939, p<0.001; 2^nd^ follow-up: ICC = 0.92; 95%CI = 0.88–0.94; p<0.001). Given the lack of previous studies identifying a cutoff for handgrip strength weakness among young pregnant women, we classified weakness according to the 20^th^ percentile of the sample distribution of handgrip strength in first assessment (i.e., ≤ 20.67 kgf represented weakness), which is common practice among epidemiological studies with other populations [19, 20].

The strength of hip adductors was assessed using the Lafayette™ manual muscle testing system (Model 01163, Lafayette Instrument Company, Lafayette, Indiana). Participants were assessed in supine position, with knees and hips flexed, and hips abducted at 30° and in neutral rotation. Three maximal voluntary isometric contractions of five seconds were requested with one minute of rest between repetitions [21]. The mean of three contractions was used for data analysis and muscle weakness classification (i.e., cutoff point of ≤ 13.8 kgf) [22].

### Data analysis

Data were analyzed using the Statistical Package for Social Sciences software (version 20.0, IBM Corp., USA). To characterize the sample, descriptive statistics were presented according to age groups (adolescents and adults), and comparisons between groups were performed using the Chi-squared test (categorical variables) and unpaired T-test (continuous variables). The Chi-square test was used to examine the association between muscle weakness at each assessment during pregnancy and postpartum and by age group (adolescent versus adult). The Mann-Whitney test was used to examine the same associations, but with continuous measures of muscle strength.

We used generalized estimating equations (GEE) to model longitudinal relationships between muscle weakness and age. GEE is an extension of the generalized linear model that considers correlations within subjects in repeated measurements, allows missing data within subjects, and is suitable for estimating population-averaged effects over time. We used an unstructured correlation framework. The first model was unadjusted. The second model adjusted for delivery type, the only variable besides education and age, for which there was a statistically significant difference between age groups. Supplemental models were conducted including adjustments for income sufficiency, race/color, BMI, and self-rated health.

## Results

Table 1 shows the sample characteristics according to age group. In addition to expected differences in mean age and educational level, a significantly higher proportion of vaginal deliveries (p < 0.005) were observed among adolescents than adults (Table 1). Other variables analyzed were not statistically different between groups.

**Table 1:** Sample characteristics according to age group.

Variables	Age groups		Total	p-value
	Adolescents (13 to 18 years)	Adults (23 to 28 years)		
	N (%) or Mean (SD)			
**Age** (years)	16.36 (± 1.32)	24.88 (± 1.48)	20.62 (±4.50)	< 0.001^a^
**Race/color**				
Black or brown	37 (74.0%)	30 (60.0%)	67 (67%)	0.137^b^
White	13 (26.0%)	20 (40.0%)	33 (33%)	
**Educational level**				
Less than elementary school	18 (36.0%)	2 (4.0%)	20 (20%)	< 0.001^b^
Elementary school or more	32 (64.0%)	48 (96.0%)	80 (80%)	
**Income sufficiency** ^d^				
Very sufficient	9 (18.0%)	12 (24.5%)	21 (21.2%)	0.715^b^
Sufficient	29 (58.0%)	27 (55.1%)	56 (56.6%)	
Insufficient	12 (24.0%)	10 (20.4%)	22 (22.2%)	
**Marital Status**				
Married or stable union	35 (70.0%)	42 (84.0%)	77 (77.0%)	0.228^b^
Dating	9 (18.0%)	4 (8.0%)	13 (13.0%)	
Without partner	6 (12.0%)	4 (8.0%)	10 (10.0%)	
**Type of delivery** ^d^				
Vaginal	27 (64.3%)	15 (34.1%)	42 (48.8%)	0.005^c^
Cesarean	15 (35.7%)	29 (65.9%)	44 (51.2%)	
**Episiotomy** ^e^				
Yes	7 (25.9%)	5 (33.3%)	12 (28.6%)	0.726^c^
No	20 (74.1%)	10 (66.7%)	30 (71.4%)	
**Days in hospital after childbirth** ^d^	4.36 (±2.88)	5.16 (± 6.92)	4.77 (±5.33)	0.488^a^
**Practiced physical activity at baseline**				
Yes	11 (22.0%)	11 (22.0%)	22 (22%)	1.000^b^
No	39 (78.0%)	39 (78.0%)	78 (78%)	
**Practiced physical activity at the 3rd trimester** ^d^				
Yes	14 (34.1%)	11 (24.4%)	25 (29.1%)	0.322^b^
No	27 (65.9%)	34 (75.6%)	61 (70.9%)	
**Self-rated health**				
Good	40 (80.0%)	36 (72.0%)	76 (76.0%)	0.349^b^
Moderate or bad	10 (20.0%)	14 (28.0%)	24 (24.0%)	
**BMI**				
Underweight	4 (8.0%)	3 (6.0%)	7 (7.0%)	0.187^c^
Eutrophic	35 (70.0%)	29 (58.0%)	64 (64.0%)	
Overweight	8 (16.0%)	17 (34.0%)	25 (25.0%)	
Obese	3 (6.0%)	1 (2.0%)	4 (4.0%)	

BMI = Body Mass Index; SD = Standard deviation.

^a^p-value for unpaired t-test.

^b^p-value for Chi-squared test.

^c^1 p-value for Fisher exact test.

^d^1 missing value for income sufficiency, 14 for physical activity at the 3rd trimester, for type of delivery and for days in hospital after childbirth.

^e^Assessed only for those who had a vaginal birth.

Table 2 shows the proportion of women with muscle weakness at different assessment points. Groups were statistically different in the third trimester for hip adductor strength, with a higher proportion of pregnant adolescents (62.5%) with muscle weakness than adults (31.8%). Results were similar when using a continuous measure of muscle strength. Median muscle strength at each time-point for both groups is presented in the S1 Table. At baseline, both groups differ statistically only in relation to handgrip strength, with adolescents being weaker than adults. In the third trimester, adolescents presented weaker hip adductor strength than adults. At this time-point, the median hip adductor strength measure for adolescents was 13.33 compared to 15.50 in adults (p-value 0.041, S1 Table). The reduction in adductor strength among adolescents from baseline to third trimester was of around 2.5Kgf, while among adults it was around 1.5Kgf. For handgrip strength, the reduction was similar for both groups (≃1.0Kgf). In the postpartum assessment, the groups did not differ significantly.

**Table 2:** Muscle weakness during pregnancy and postpartum in adolescents (13 to 18 years) and adults (23 to 28 years).

	Age groups	Handgrip strength		Hip adductor strength	
		Normal	Muscle weakness	Normal	Muscle weakness
**Until the 16th gestational week**	**Adolescents**	37 (74.0%)	13 (26.0%)	39 (78.0%)	11 (22.0%)
	**Adults**	42 (84.0%)	8 (16.0%)	42 (84.0%)	8 (16.0%)
	p-value	0.22		0.44	
**3rd trimester**	**Adolescents**	29 (72.5%)	11 (27.5%)	**15 (37.5%)**	**25 (62.5%)**
	**Adults**	34 (79.1%)	9 (20.9%)	**30 (68.2%)**	**14 (31.8%)**
	p-value	0.48		**0.005**	
**Between 4th and 6th week postpartum**	**Adolescents**	27 (67.5%)	13 (32.5%)	13 (35.1%)	24 (64.9%)
	**Adults**	35 (81.4%)	8 (18.6%)	14 (35.0%)	26 (65.0%)
	p-value	0.15		0.99	

**Fig 2:**
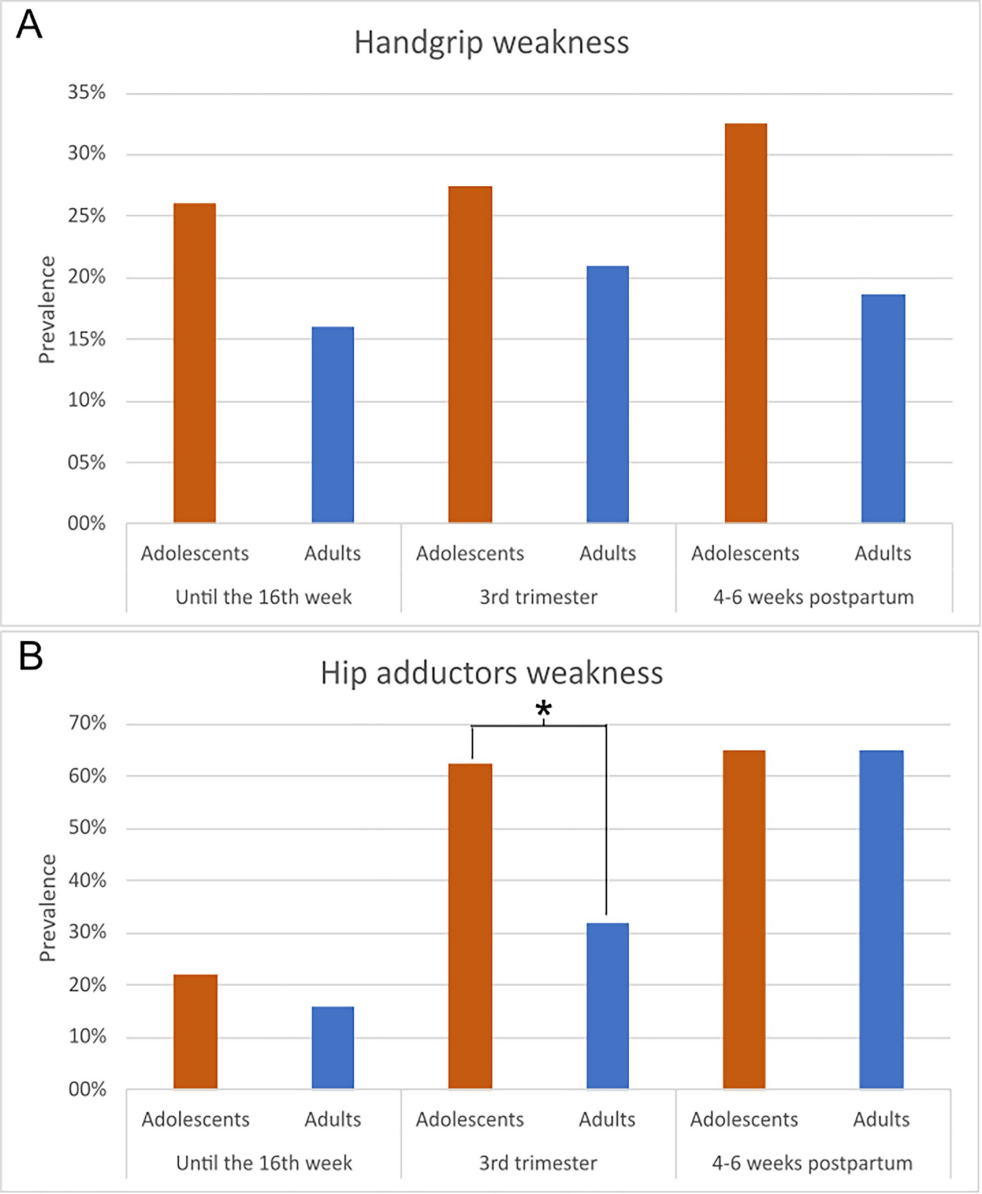
A. Proportion of women with handgrip weakness; B. Proportion of women with hip adductor weakness. (N = 100). *p = 0.005.

Fig 2 depicts the proportion of participants with handgrip and hip adductor weakness across assessments. More adolescents presented handgrip weakness in all assessments than adults (Fig 2A). Muscle weakness also increased from first to third assessment among adolescents, whereas for adults it slightly increased between first and second assessments and declined from second to third assessment.

More pregnant adolescents had hip adductor weakness in the first and second assessments compared to adults; however, prevalence was similar between groups in the third assessment (Fig 2B). For both adolescents and adults, muscle weakness increased substantially from the first to third assessment. Regarding the difference between the groups, statistical significance was observed only for the hip adductor muscles in the third trimester of pregnancy (p-value = 0.005).

Table 3 shows the GEE results for muscle weakness according to age group and assessments. Pregnant adolescents were 2.1-fold more likely to present hip adductor weakness (p = 0.07) than adults, but the result was not statistically significant ⍺<0.05. Participants were 4.35 and 9.45 more likely to present hip adductor weakness in the third trimester and during postpartum, respectively, than before the 16^th^ gestational week. No statistically significant results for handgrip weakness were found. Results were similar when the models were adjusted for other study variables (income sufficiency, race/color, BMI, or self-rated health), as presented in the supporting information files (S2–S4 Tables). For the model adjusting for self-rated health, the difference between adolescents and adults for hip adductor weakness was statistically significant (OR = 2.20; 95%CI: 1.00; 4.82, p = 0.05).

**Table 3:** Generalized estimating equations for longitudinal relationships between muscle weakness and age according to follow-up assessments (assessment until the 16th gestational week and adult group were reference categories).

	Handgrip weakness				Hip adductor weakness			
	Model 1		Model 2		Model 1		Model 2	
	OR (95% CI)	p	OR (95% CI)	p	OR (95% CI)	p	OR (95% CI)	p
**Age groups**								
Adults	1		1		1		1	
Adolescents	1.78 (0.74; 4.24)	0.20	1.91 (0.74; 4.93)	0.18	1.81 (0.89; 3.71)	0.10	2.11 (0.95; 4.68)	0.07
**Time of assessment**								
Until the 16th week	1		1		1		1	
3rd trimester	1.21 (0.77; 1.91)	0.41	1.31 (0.84; 2.05)	0.24	3.84 (2.21; 6.66)	<0.001	4.35 (2.43; 7.80)	<0.001
4–6 weeks postpartum	1.29 (0.81; 2.05)	0.28	1.39 (0.88; 2.21)	0.16	8.32 (4.51; 15.36)	<0.001	9.45 (4.92; 18.15)	<0.001
**Cesarean Section**								
No	-		1		-		1	
Yes	-		1.49 (0.59; 3.73)	0.40	-		1.46 (0.66; 3.24)	0.35

Note: Model 1 considers age group and time of assessment as predictors of muscle weakness. Model 2 considers age group, time of assessment and mode of delivery. OR: Odds Ratio.

## Discussion

This study investigated the prevalence of hip adductor and handgrip weakness during pregnancy and early postpartum among adolescents and adults. Muscle weakness was more common in pregnant adolescents than adults. However, differences were only statistically significant for hip adductors in the third gestational trimester.

Regarding hip adductor strength, both groups had similar proportions of pregnant women with muscle weakness, being smaller in the first (until the 16^th^ gestational week) than in the second (third gestational trimester) and third assessments (between fourth and sixth week after delivery). GEE results indicated significantly higher chances of hip adductor weakness in the third trimester and postpartum than in the 16^th^ gestational week, regardless of age group. Although the prevalence of handgrip weakness increased between assessments, differences were not statistically significant.

Other studies with adult women also reported some changes in muscle strength of upper and lower limbs during pregnancy [23, 24]. Similar to the present results, one study described a small non-significant loss in handgrip strength in the late stage compared to the middle stage of pregnancy [23]. Another study [24] also found a reduction in muscle strength in both upper and lower limbs from pre-pregnancy to 6 weeks postpartum, but a greater reduction in the lower body than the upper body. The authors discussed that this was contrary to what would be expected with gestational weight gain, i.e., the maintenance of lower limb strength in response to the greater body load. They also observed that muscle strength was not entirely recovered even at six months postpartum [24]. Another exploratory study with 19 pregnant women and 15 non-pregnant controls reported changes in the muscle architecture, including increasing in thickness from early to late pregnancy, but no changes in muscle strength [25]. The increase in the water content of the muscles during pregnancy reported in the same study helps explain why the increase in muscle thickness was not followed by the increase in muscle strength as would be expected. This may lead to skeletal muscles constantly working closer to their maximal capacity during antigravitational movements. These factors, associated with the increased mechanical pressure on the pelvis and hips, and elevated progesterone and relaxin levels that increase joint laxity may explain some effects in the musculoskeletal system among pregnant women, such as joint pain, reduced postural stability and increased risk of injuries [26].

Results of the present study were significant only for hip adductors, probably because these muscles are more impacted than upper limbs muscles during pregnancy and postpartum. Even though handgrip strength is an important indicator of health and functional capacity [27], it may be less affected by body weight gain during pregnancy than hip muscles when considering the influence of body mass on trunk weight distribution and gait kinematics. A previous study with pregnant women evaluated in the third gestational trimester and one year after delivery observed that walking during pregnancy increased the demand for lower limb muscles, especially hip abductors [28]. This may help explain the hip adductor finding, since overload of a given muscle may impact the strength of its antagonists.

Two hypotheses may explain the worse results for hip adductor strength among adolescents compared to adults. Adolescents present less muscle, joint, and bone development, especially in the pelvis [29]. Thus, they may go into pregnancy with less muscular reserve than adults and, therefore, be more susceptible to reach the criteria for weakness. In fact, median hip adductor strength was lower among adolescents in the baseline assessment than among adults; although, the results failed to reach statistical significance. Additionally, it is possible that adolescents lose more strength than adults throughout pregnancy, because their bodies are not fully prepared to support the changes of pregnancy. Muscle strength seemed to decline more from early to late pregnancy among adolescents than adults in our study, as reported in the S1 Table. Considering that adolescence is the phase of growth and final pubertal maturation, pregnancy may lead to a situation in which mother and child compete for nutrients [30]. This in turn may decrease the physiological reserves of the maternal body and increase muscle weakness [31]. Across the life-course, muscle weakness initiated during adolescent pregnancy and postpartum may negatively affect muscle strength with age, corroborating studies that observed low physical function in middle-aged [10] and older adult [3] women who were once adolescent mothers. Given the importance of muscle strength to physical functioning, this study provides novel insights into pathways between pregnancy during adolescence and worse physical function over the long term.

Weakness in the muscles around the hips, including the hip adductor, has been found to be associated with the presence and severity of pelvic girdle pain related to pregnancy [22, 32]. Thus, together with previous literature, our results support the importance of assessing adductor muscle strength during pregnancy for all women, especially among adolescents, to detect early those at higher risk of physical and painful disorders. They also support the importance of engaging in resistance training during pregnancy to mitigate the negative effects of pregnancy on lower limbs muscle strength. In this study, less than 30% of the participants reported regular practice of any kind of physical activity during the third trimester of pregnancy, with no differences between adolescents and adults. This indicates the need for health promotion efforts to increase awareness of the importance of physical activity during pregnancy.

Adolescents and adults in this study did not differ statistically for most of the socioeconomic and health-related variables presented in Table 1. With the exception of obvious expected differences in mean age and education, the two groups differed only in relation to type of delivery. Cesarean section was significantly higher among adults (65.9%) than adolescents (35.7%). Since it may impact lower extremity muscle strength, particularly in the postpartum assessment, it was added to the regression models. The high prevalence of this type of delivery in our sample corroborates other studies showing high cesarean birth rates in Brazilian women [33]. During 2014 to 2017, 55.8% of births in Brazil were performed via Cesarean section, which is much higher than the global rate (18.6%) [34]. Additionally, almost half of births among those with favorable initial conditions for vaginal delivery were Cesarean sections, which suggests that there is no clinical basis for such a high percentage of surgeries. The high rate of this type of procedure is higher among those with better education and socioeconomic conditions [35, 36] and among the births occurring in the private health sector compared to the public sector [33]. It has been suggested that the women’s fears of the physiological consequences of vaginal delivery and their desire to keep their sexual performance intact are some of the reasons for these higher rates [37]. Opting for a Cesarean section may be less common among vulnerable groups such as low-income adolescents, which may help explain the lower rate of surgeries among this group compared to the adult group.

The study had some limitations. The relatively small sample size might influence the study power and ability to detect associations. For example, at all assessments, more adolescents than adults had handgrip weakness, but these differences were not statistically significant. In addition, the study included only primiparous women, and results could be different if the pregnant women already had children, especially among the adolescents. The measure of physical activity did not consider type and frequency of the exercise, which could impact the results. However, the time spent with leisure physical activities, including resistance training, as well as with activities such as walking or biking, was very low in both samples, with no differences among both age groups and slightly lower engagement in the third trimester (data upon request). The study also has important strengths, including the use of objective physical function measures, extensive socio-demographic measures, and high participant retention over time.

## Conclusions

The proportion of adolescent and adult pregnant women with muscle weakness (especially hip adductors) increased from the first weeks of pregnancy to the third trimester and postpartum period. A significantly higher proportion of hip adductor weakness was identified among adolescents in the third gestational trimester compared to adults. These findings highlight the importance of investigating muscle strength during pregnancy to identify women who may need additional support, such as physical therapy, to regain and maintain muscle strength.

## Supporting information

S1 Table Comparison of continuous muscle strength measures between adolescents (13 to 18 years) and adults (23 to 28 years). (DOCX)

S2 Table Generalized estimating equations for longitudinal relationships between muscle weakness and age according to follow-up assessments, adjusted for mode of delivery and body index mass. (DOCX)

S3 Table Generalized estimating equations for longitudinal relationships between muscle weakness and age according to follow-up assessments, adjusted for mode of delivery and income sufficiency. (DOCX)

S4 Table Generalized estimating equations for longitudinal relationships between muscle weakness and age according to follow-up assessments, adjusted for mode of delivery and race/color. (DOCX)

S5 Table Generalized estimating equations for longitudinal relationships between muscle weakness and age according to follow-up assessments, adjusted for mode of delivery and self-rated health. (DOCX)

S1 Data (SAV)
