# Supplementary material for: Muscle strength during pregnancy and postpartum in adolescents and adults
Source: PLoS One. 2024 Mar 27;19(3):e0300062. doi: 10.1371/journal.pone.0300062 (PMC10971575; doi:10.1371/journal.pone.0300062)
Supplement: S1 Table — (DOCX) [file pone.0300062.s001.docx]

**S1 Table 1: Comparison of continuous muscle strength measures between adolescents (13 to 18 years) and adults (23 to 28 years).**

| Age groups | Handgrip strength | Hip adductor strength |
| --- | --- | --- |
|  | Median (IQR) | |
|  | **Until the 16th gestational week** | |
| Adolescents | 23.33 (7.00) | 15.83 (3.73) |
| Adults | 26.67 (6.92) | 16.98 (5.67) |
| p-value | 0.028 | 0.092 |
|  | **3rd trimester** | |
| Adolescents | 24.33 (6.58) | 13.33 (5.50) |
| Adults | 25.67 (8.33) | 15.50 (5.58) |
| p-value | 0.150 | 0.041 |
|  | **Between 4th and 6th week postpartum** | |
| Adolescents | 24.00 (8.25) | 12.67 (4.17) |
| Adults | 24.00 (7.33) | 12.33 (5.25) |
| p-value | 0.394 | 0.350 |

IQR: Interquartile range.
