# Supplementary material for: Muscle strength during pregnancy and postpartum in adolescents and adults
Source: PLoS One. 2024 Mar 27;19(3):e0300062. doi: 10.1371/journal.pone.0300062 (PMC10971575; doi:10.1371/journal.pone.0300062)
Supplement: S2 Table — (DOCX) [file pone.0300062.s002.docx]

**S2 Table: Generalized estimating equations for longitudinal relationships between muscle weakness and age according to follow-up assessments, adjusted for mode of delivery and body index mass.**

|  | **Handgrip weakness** | | **Hip adductor weakness** | |
| --- | --- | --- | --- | --- |
|  | OR (95% CI) | p | OR (95% CI) | p |
| **Age groups** |  |  |  |  |
| Adults | 1 |  | 1 |  |
| Adolescents | 1.48 (0.57; 3.87) | 0.42 | 2.15 (0.97; 4.75) | 0.06 |
| **Time** |  |  |  |  |
| Until the 16th week | 1 |  | 1 |  |
| 3rd trimester | 1.34 (0.85; 2.11) | 0.21 | 5.22 (2.78; 9.83) | <0.001 |
| 4-6 weeks postpartum | 1.42 (0.88; 2.29) | 0.15 | 11.54 (5.69; 23.40) | <0.001 |
| **Cesarean section** |  |  |  |  |
| No | 1 |  | 1 |  |
| Yes | 1.43 (0.57; 3.60) | 0.45 | 1.68 (0.76; 3.71) | 0.20 |
| **Body mass index** |  |  |  |  |
| Underweight | 1.10 (0.27; 4.53) | 0.89 | 9.15 (1.78; 47.112) | 0.01 |
| Normal weight | 1 |  | 1 |  |
| Overweight | 0.24 (0.06; 0.91) | 0.04 | 0.78 (0.34; 1.80) | 0.56 |
| Obese | 2.43 (0.30; 19.72) | 0.41 | 0.98 (0.15; 6.95) | 0.98 |
