# Supplementary material for: Muscle strength during pregnancy and postpartum in adolescents and adults
Source: PLoS One. 2024 Mar 27;19(3):e0300062. doi: 10.1371/journal.pone.0300062 (PMC10971575; doi:10.1371/journal.pone.0300062)
Supplement: S3 Table — (DOCX) [file pone.0300062.s003.docx]

**S3 Table: Generalized estimating equations for longitudinal relationships between muscle weakness and age according to follow-up assessments, adjusted for mode of delivery and income sufficiency.**

|  | **Handgrip weakness** | | **Hip adductor weakness** | |
| --- | --- | --- | --- | --- |
|  | OR (95% CI) | p | OR (95% CI) | p |
| **Age groups** |  |  |  |  |
| Adults | 1 |  | 1 |  |
| Adolescents | 1.77 (0.69; 4.50) | 0.23 | 2.01 (0.89; 4.56) | 0.09 |
| **Time** |  |  |  |  |
| Until the 16th week | 1 |  | 1 |  |
| 3rd trimester | 1.30 (0.83; 2.05) | 0.25 | 4.43 (2.43; 8.07) | <0.001 |
| 4-6 weeks postpartum | 1.41 (0.88; 2.25) | 0.15 | 9.55 (4.91; 18.55) | <0.001 |
| **Cesarean section** |  |  |  |  |
| No | 1 |  | 1 |  |
| Yes | 1.36 (0.54; 3.40) | 0.52 | 1.43 (0.65; 3.14) | 0.38 |
| **Income sufficiency** |  |  |  |  |
| Very sufficient | 1 |  | 1 |  |
| Sufficient | 1.79 (0.44; 7.30) | 0.41 | 1.97 (0.70; 5.48) | 0.20 |
| Insufficient | 1.12 (0.24; 5.26) | 0.89 | 1.49 (0.46; 4.83) | 0.51 |
