# Supplementary material for: Muscle strength during pregnancy and postpartum in adolescents and adults
Source: PLoS One. 2024 Mar 27;19(3):e0300062. doi: 10.1371/journal.pone.0300062 (PMC10971575; doi:10.1371/journal.pone.0300062)
Supplement: S4 Table — (DOCX) [file pone.0300062.s004.docx]

**S4 table: Generalized estimating equations for longitudinal relationships between muscle weakness and age according to follow-up assessments, adjusted for mode of delivery and race/color.**

|  | **Handgrip weakness** | | **Hip adductor weakness** | |
| --- | --- | --- | --- | --- |
|  | OR (95% CI) | p | OR (95% CI) | p |
| **Age groups** |  |  |  |  |
| Adults | 1 |  | 1 |  |
| Adolescents | 2.07 (0.78; 5.48) | 0.14 | 2.09 (0.92; 4.75) | 0.08 |
| **Time** |  |  |  |  |
| Until the 16th week | 1 |  | 1 |  |
| 3rd trimester | 1.32 (0.84; 2.08) | 0.24 | 4.35 (2.43; 7.80) | <0.001 |
| 4-6 weeks postpartum | 1.39 (0.87; 2.21) | 0.17 | 9.46 (4.94; 18.13) | <0.001 |
| **Cesarean section** |  |  |  |  |
| No | 1 |  | 1 |  |
| Yes | 1.50 (0.59; 3.84) | 0.40 | 1.46 (0.66; 3.23) | 0.35 |
| **Race/color** |  |  |  |  |
| Black or brown | 0.64 (0.25; 1.63) | 0.35 | 1.06 (0.45; 2.50) | 0.89 |
| White | 1 |  | 1 |  |
