# Supplementary material for: Muscle strength during pregnancy and postpartum in adolescents and adults
Source: PLoS One. 2024 Mar 27;19(3):e0300062. doi: 10.1371/journal.pone.0300062 (PMC10971575; doi:10.1371/journal.pone.0300062)
Supplement: S5 Table — (DOCX) [file pone.0300062.s005.docx]

**S5 table: Generalized estimating equations for longitudinal relationships between muscle weakness and age according to follow-up assessments, adjusted for mode of delivery and self-rated health.**

|  | **Handgrip weakness** | | **Hip adductor weakness** | |
| --- | --- | --- | --- | --- |
|  | OR (95% CI) | p | OR (95% CI) | p |
| **Age groups** |  |  |  |  |
| Adults | 1 |  | 1 |  |
| Adolescents | 1.97 (0.76; 5.09) | 0.16 | 2.20 (1.00; 4.82) | 0.05 |
| **Time** |  |  |  |  |
| Until the 16th week | 1 |  | 1 |  |
| 3rd trimester | 1.36 (0.86; 2.14) | 0.19 | 4.64 (2.57; 8.37) | <0.001 |
| 4-6 weeks postpartum | 1.45 (0.90; 2.33) | 0.13 | 10.22 (5.25; 19.90) | <0.001 |
| **Cesarean section** |  |  |  |  |
| No | 1 |  | 1 |  |
| Yes | 1.52 (0.60; 3.83) | 0.38 | 1.50 (0.68; 3.33) | 0.32 |
| **Self-rated health** |  |  |  |  |
| Good | 0.75 (0.32; 1.78) | 0.51 | 0.63 (0.27; 1.48) | 0.29 |
| Moderate or bad | 1 |  | 1 |  |
